# Stimulation of Metabolic Activity and Cell Differentiation in Osteoblastic and Human Mesenchymal Stem Cells by a Nanohydroxyapatite Paste Bone Graft Substitute

**DOI:** 10.3390/ma15041570

**Published:** 2022-02-19

**Authors:** Carolina Herranz-Diez, Aileen Crawford, Rebecca L. Goodchild, Paul V. Hatton, Cheryl A. Miller

**Affiliations:** 1 Unit of Biophysics and Bioengineering, School of Medicine and Health Sciences, University of Barcelona, Casanova 143, 08014 Barcelona, Spain; carolinaherranz@ub.edu; 2 School of Clinical Dentistry, University of Sheffield, Claremont Crescent, Sheffield S10 2TA, UK; paul.hatton@sheffield.ac.uk (P.V.H.); c.a.miller@sheffield.ac.uk (C.A.M.); 3 Ceramysis Ltd., 914 Herries Road, Sheffield S6 1QW, UK; b.hutchinson@ceramisys.com

**Keywords:** bone graft substitute, nanohydroxyapatite, osteoblastic cells, osteoblasts, osteogenic differentiation, calcium-sensing receptor, MSCs, cell activity

## Abstract

Advances in nanotechnology have been exploited to develop new biomaterials including nanocrystalline hydroxyapatite (nHA) with physical properties close to those of natural bone mineral. While clinical data are encouraging, relatively little is understood regarding bone cells’ interactions with synthetic graft substitutes based on this technology. The aim of this research was therefore to investigate the in vitro response of both osteoblast cell lines and primary osteoblasts to an nHA paste. Cellular metabolic activity was assessed using the cell viability reagent PrestoBlue and quantitative, real-time PCR was used to determine gene expression related to osteogenic differentiation. A potential role of calcium-sensing receptor (CaSR) in the response of osteoblastic cells to nHA was also investigated. Indirect contact of the nHA paste with human osteoblastic cells (Saos-2, MG63, primary osteoblasts) and human bone marrow-derived mesenchymal stem cells enhanced the cell metabolic activity. The nHA paste also stimulated gene expression of runt-related transcription factor 2, collagen 1, alkaline phosphatase, and osteocalcin, thereby indicating an osteogenic response. CaSR was not involved in nHA paste-induced increases in cellular metabolic activity. This investigation demonstrated that the nHA paste has osteogenic properties that contribute to clinical efficacy when employed as an injectable bone graft substitute.

## 1. Introduction

The inorganic phase of bone is composed of carbonated apatite that is generally nanoscale and amorphous when first formed [1,2]. Synthetic hydroxyapatite (HA) has been used extensively as a bone graft substitute, where it promotes healing via osteoconduction [3]. Despite some similarities to bone mineral, macroscale HA resorbs slowly if at all, and it is a brittle material [4,5]. The emergence of nanotechnology has enabled the development of nanoscale hydroxyapatites (nHA) [6,7] which have a crystal structure closer to that of native bone. There are various chemical methods for the manufacture of nHA particles including sol–gel and hydrothermal syntheses, emulsion-based methods, microwave processing, solid-state methods, and wet precipitation methods (reviewed in [8,9]). Depending on the method used, the resultant HA particles can differ in size, shape, chemical composition, and crystallinity, which will influence the rate of resorption and the biological response [10]. Nanostructured HA particles are most commonly produced using wet precipitation methods and remain unsintered, which, in combination with the high surface area of these materials, results in the bioresorbability being closer to that of the natural HA in bone [11,12,13]. A further advantage of nanoscale HAs is their increased bioactivity compared to macroscale HA [14,15]. The increased surface area and roughness of nHA also enhance cell adhesion and cell–matrix interaction [9]. Additionally, there is some evidence that bioinspired nHAs are superior to block/particulate HA when used as bone graft substitutes, with both clinical and in vivo studies reporting that nHA promotes bone-tissue regeneration [12,16,17,18].

There are some concerns regarding potential cytotoxicity of nHA due to cellular internalisation of the nanoparticles, which is a general phenomenon seen with nanoparticles of other materials [19,20]. nHA particles are usually internalised by pinocytosis, endocytosis, or phagocytosis [21]. Cell internalisation of nHA is dependent on the physicochemical properties [21,22], particle size and shape [23,24,25,26,27], charge [28], cell type, and uptake mechanism [21]. Intracellular nHA particles are eventually degraded in endolysosomes by the acidic environment of the organelles [29,30]. In vitro investigations have reported that needle-shaped and short-rod-like forms of nHA and particles with small (20 nm) diameters may induce cytotoxic effects in vitro such as apoptosis of osteoblasts [31] and other cell types or inhibition of cell growth in vitro [32]. Additionally, in vitro exposure of macrophages to high levels of nHA caused cell death due to high intracellular calcium levels caused by Ca^2+^ release from intracellular nHA particles which disrupted the intracellular Ca^2+^ homeostasis [22]. Several in vitro studies have raised concerns that increasing exposure times to nanoparticles of HA could promote cytotoxicity [33,34,35,36]. However, internalisation of nHA particles by osteoblastic cells does not necessarily cause cytotoxicity or affect their normal cell behavior [37].

The cellular mechanisms by which the nHAs exert their osteogenic effects are still unclear, but one candidate mechanism may involve the calcium-sensing receptor (CaSR) [38]. Ca^2+^ ions are critical for a diverse range of biological functions, including intracellular signal transduction, mineralisation of bone matrix, and hormone secretion. To enable these functions to occur, the extracellular Ca^2+^ concentration is very tightly controlled and the CaSR is an important regulator in maintaining calcium homeostasis [39]. However, the CaSR is also known to have biological roles outside its role in calcium regulation [38], including an important role in bone development, mineralization, and turnover [40]. Functional CaSRs are found on cells of the osteoblastic lineage, osteoclasts, and bone marrow mesenchymal cells (BM-MSCs) [40,41], which are precursor cells of the osteoblastic cell lineage; MG63 and Saos-2 cells also have functional CaSRs [42,43]. The CaSR senses perturbations in local extracellular Ca^2+^ concentrations. A rise in the extracellular Ca^2+^ will activate the receptor initiating intracellular signaling pathways to modulate the function of the cell [39]. The CaSR has an important role in cell proliferation [44] and cell migration [45] and modulates osteoinduction [46] by regulating the recruitment, differentiation, and survival of osteoblasts and osteoclasts [47,48]. An important feature of the CaSR is its biased agonism [49]. This means that different ligands can activate the receptor, causing differing effects on the intracellular signaling pathways. Davey et al. [50] studied the allosteric effects of the calcimimetics cinacalcet and NPS-R568, and the calcilytic NPS-2143 on Ca^2+^-mediated signaling across multiple pathways and demonstrated that these ligands mediate their effect on the CaSR. Cinacalcet (3-(trifluoromethyl) cinnamic acid) is a CaSR agonist that has been used clinically to reduce parathyroid hormone (PTH) release in the treatment of hyperparathyroidism [51]. NPS 2143 is a CaSR antagonist which inhibits the CaSR [52,53].

To summarise, nHA shows great promise as a bone graft substitute, supported by clinical data from the first generation of medical devices based on this technology. However, little is understood regarding the interaction of bone cells with these novel materials, including how these interactions contribute to tissue regeneration. The aim of this study was, therefore, to investigate the cellular response of osteoblastic cells to nHA paste and explore whether the osteoblast CaSR was involved in the response of the cells to nHA. A water-based, non-setting, nHA paste was used for this investigation where four different bone cells were selected to interrogate different aspects of interaction. The cellular response was first assessed using the human bone-derived cell lines (Saos-2 and MG63) with different osteoblastic maturation states (Saos-2 cells have a mature osteoblastic phenotype while MG63 cells have an immature osteoblastic phenotype). The effect of nHA paste on the cellular response of primary human osteoblasts (HOBs) and human bone marrow-derived mesenchymal stem cells (hBM-MSCs) was also assessed. The Saos-2 and MG63 cell lines were originally derived from human primary osteosarcomas. Both Saos-2 and MG63 cells express osteoblastic characteristics [54,55] and have been extensively used as models of osteoblastic cells in the bone research field [56]. The effects of a CaSR agonist and an antagonist on MG63 cells were investigated to determine whether the nHA paste interacted with the CaSR.

## 2. Materials and Methods

The nHA paste used in these experiments was based on a commercial nHA paste (ReproBone novo, Ceramisys Ltd., Sheffield, UK). The nHA was a suspension of hydroxyapatite (Ca_10_(PO_4_)_6_(OH)_2_) nanoscale particles (40–60 nm) at a concentration of 38 wt.% in water which was produced by a reverse osmosis process. The authors have previously described the synthesis [12] and physicochemical properties of this nHA paste [57]. The nHA was composed of a pure HA phase with low crystallinity, and rod-shaped HA particles. The nHA paste was a stable colloidal system in which no dispersant materials or solvents were needed or included. To aid physical handling, the paste was dispensed into 2.5 mL syringes. The rheological properties were determined to be like those of shear-thinning, non-Newtonian liquids.

### 2.1. Preparation of nHA Paste-Conditioned Culture Medium Extract

Samples of nHA paste 1 cm in length were expelled from the syringe and incubated in 1 mL of culture medium (CM) at 37 °C in a humidified atmosphere of 95% air with 5% CO_2_ for 24 h. The media in contact with the paste were then removed and either (i) centrifuged at 1,000 g for 5 min (paste-conditioned medium 1000 (PCM-1000)) for 5 min, (ii) centrifuged at 12,000 g for 5 min (paste-conditioned medium 1200 (PCM-1200)), or (iii) filtered through a 0.22 µm membrane (paste-conditioned medium 0.22 (PCM-0.22)). Paste-conditioned media were prepared freshly for each experiment.

### 2.2. Cell Culture

Different commercially obtained human osteoblastic cell types were used in this study. All cell types were cultured at 37 °C in a humidified atmosphere of 95% air and 5% CO_2_ using standard tissue culture procedures. Concentrations of additives given below in the culture media are the final concentrations used for experiments.

***Human osteosarcoma-derived cell lines, MG63 and Saos-2:*** MG63 cells (purchased from Sigma-Aldrich, Gillingham, UK) were cultured in Eagle’s Modified Minimal Essential Medium (EMEM, with Earle’s salts, L-glutamine, sodium bicarbonate, and sterile-filtered liquid, purchased from Sigma Aldrich, Gillingham, UK) supplemented with 10% foetal bovine serum (FBS, non-USA origin, heat-inactivated, suitable for cell culture, purchased from Sigma-Aldrich, Gillingham, UK); penicillin and streptomycin antibiotic 50 U/mL and 50 µg/mL, respectively (purchased from Sigma-Aldrich, Gillingham, UK, as a stabilised stock solution, with 50,000 U penicillin and 50,000 µg streptomycin/mL, 0.1 µ filtered bioreagent suitable for cell culture); 1% nonessential amino acids (NEAA); and 2 mM L-glutamine. Saos-2 cells (purchased from Sigma-Aldrich, Gillingham, UK) were cultured in McCoy’s 5A modified culture medium (McCoy’s 5A modified culture medium with high glucose of 3 g/L, L-glutamine, and bacto-peptone, without sodium pyruvate and HEPES, purchased from Thermo Fisher Scientific, Paisley, UK) supplemented with 10% FBS and penicillin and streptomycin at 50 U/mL and 50 µg/mL, respectively.

***Human primary osteoblasts (HOBs):*** Primary HOBs were purchased from PromoCell, Heidleberg, Germany, and cultured in proprietary Osteoblast Growth Medium (Osteoblast Growth Medium (ready to use), with 0.1 mL foetal calf serum/mL medium, PromoCell, Heidleberg, Germany). The osteoblast growth medium was supplemented with penicillin and streptomycin at 50 U/mL and 50 µg/mL, respectively. The HOBs were used for experimentation up to passage 5.

***Bone marrow human mesenchymal stem cells (hBM-MSCs):*** Primary hBM-MSCs purchased from PromoCell, Heidleberg, Germany, were cultured in proprietary StemMACS MSC expansion media (StemMACS Expansion Media human with L-glutamine, foetal bovine serum, phenol red, Miltenyi Biotech Bisley, UK) containing 50 U/mL penicillin and 50 µg/mL streptomycin. The cells were cultured to passage 5.

***Murine myoblast cell line:*** The C2C12 murine myoblast cell line was purchased from Sigma-Aldrich, UK. The cells were cultured in Dulbecco’s Modified Eagle’s medium, high glucose formula (DMEM, 4.5 g/L of glucose, L-glutamine, sodium bicarbonate, without sodium pyruvate, sterile-filtered liquid, Sigma Aldrich, Gillingham, UK) supplemented with 5% FBS, 50 U/mL penicillin, and 50 µg/mL streptomycin [58].

### 2.3. Cell Viability Assays

The biocompatibility of the nHA paste on hBM-MSCs and bone-derived cells was assessed by indirect contact assay based on the “Biological Evaluation of Medical Devices—Part 5: Tests for *In Vitro* Cytotoxicity ISO 10993-5:2009” international standard guidelines and test systems for the assessment of cytotoxicity in medical devices [36]. Briefly, cells were seeded at 50,000 cells/well in 24-well culture plates and incubated for 24 h at 37 °C in a humidified atmosphere of 95% air with 5% CO_2_. The culture medium was removed and replaced with the HA paste-conditioned culture medium (refer above), and the cells were incubated with nHA paste-conditioned media for 24 h at 37 °C. Cell viability was assessed using the vital dye resazurin which can diffuse into cells and is reduced in the cell cytoplasm and mitochondria to the fluorescent compound resorufin which diffuses out of the cell and can be detected by measuring the fluorescence of the culture media. A commercial preparation of resazurin dye (PrestoBlue™, Thermo Fisher Scientific, Paisley, UK) was used to assess cell viability in this study. After incubation with nHA paste-conditioned media, PrestoBlue end-point analyses were conducted according to the manufacturer’s instructions. In brief, PrestoBlue™ was added to 10% (*v*/*v*) in the medium of the cell cultures and incubated at 37 °C for 1 h. To assess any noncellular reduction of the resazurin dye (reagent controls), PrestoBlue™ was added to 10% (*v*/*v*) to the cell culture medium alone (no cells present) and incubated in the same way as for the cell cultures. After incubation with PrestoBlue™, 200 µL samples of the culture media and reagent controls were transferred to a 96-well plate, and the fluorescence intensity of the culture media was measured (excitation wavelength 560 nm, emission wavelength 590 nm) using a Tecan plate reader (Tecan Infinite 200, Tecan, Männedorf, Switzerland). The mean fluorescence of the reagent controls was subtracted from the fluorescence reading from each cell culture.

### 2.4. Determination of the Ca^2+^ Ions Released from the nHA Paste

***Ca^2+^ colorimetric assay****:* To measure the free calcium concentration in the PCM, the Calcium Colorimetric Assay Kit (Sigma-Aldrich, Gillingham, UK) was used. The linear range of detection for calcium ions in this assay is between 0.4 and 2.0 μg. The calcium ion concentration was determined by measuring the chromogenic complex formed between calcium ions and the *o*-cresolphthalein. The chromogenic complex was measured spectrophotometrically by determining the optical density (OD) at 575 nm. The OD was proportional to the concentration of calcium ions present. PCM was prepared as described above, and MilliQ water and culture medium were used as negative and positive control, respectively. The assays were conducted following the manufacturer’s instructions. Briefly, calcium standards were prepared with the following calcium concentrations: 0.4, 0.8, 1.2, 1.6 and 2.0 μg/50 µL. Fifty microlitre samples of PCM or the calcium standards were added to wells of a 96-well plate. Ninety microlitres of Chromogenic Reagent and 60 μL of the Calcium Assay Buffer were added to each of the wells containing standards, samples, or controls. The assay plate was protected from light and incubated for 10 min at room temperature. The absorbance was measured at 575 nm using a plate reader (Tecan 200 Infinite, Tecan, Männedorf, Switzerland).

***Ca^2+^ and Na^+^ ion determination by inductively coupled plasma optical emission spectroscopy (ICP-OES):*** Samples of nHA paste-conditioned medium and culture medium were analysed by ICP-OES by Sheffield Analytical Services, Sheffield Assay Office, Sheffield, UK, using standardised proprietary methods.

### 2.5. Study of the Effect of Ions Released from the Paste on Cell Metabolism and Their Potential Mechanism

***Effect of low concentrations of Ca^2+^ and PO_4_ ^3−^ on cell metabolism****:* A stock solution of 100 mM CaCl_2_ was made in distilled water and further diluted in Ca^2+^-free Minimal Essential Eagle’s Joklik Modification medium (MEM Joklik medium, Minimal Essential Medium Eagle with Joklik Modification, L-glutamine, without calcium chloride and sodium bicarbonate, suitable for cell culture, Sigma-Aldrich, Gillingham, UK) to obtain the required concentrations of Ca^2+^ ions. To obtain the PO_4_^3−^ ion concentrations, two phosphate solutions at 100 mM were used: Na_2_HPO_4_ and Na_2_H_2_PO_4_, following the Cold Spring Harbor Protocols [59] to obtain a sodium phosphate buffer (pH 7). Both stock solutions were used at final concentrations of 1.5 mM, 1 mM, 0.5 mM, 0.25 mM, and 0.1 mM using Ca^2+^-free medium (Minimum Essential Medium Eagle Joklik Modification). MG63 cells were seeded in MEM with 10% FBS into a 24-well culture plate at a concentration 50,000 cells/well and incubated at 37 °C. After 24 h, the medium was removed and the cell layers washed with Dulbecco’s phosphate-buffered saline (PBS, modified, without calcium chloride and magnesium chloride, liquid, sterile-filtered, suitable for cell culture, Sigma-Aldrich, Gillingham, UK). The PBS was removed and replaced with MEM Joklik medium containing the described concentrations of CaCl_2_ and the phosphate buffer as appropriate. The cells were incubated for a further 24 h at 37 °C after which, end-point PrestoBlue™ analyses were conducted to determine cell viability as described above.

### 2.6. CaSR Agonist, 3-(Trifluoromethyl) Cinnamic Acid

3-(Trifluoromethyl) cinnamic acid, Cinacalcet, was prepared at a stock concentration of 20 mM in ethanol. Final assay concentrations of (i) 100 nM, (ii) 250 nM, (iii) 1000 nM, (iv) 10000 nM, and (v) 25000 nM were prepared by dilution of the stock solution in control or in paste-conditioned culture media. Briefly, 5 × 10^4^ MG63 cells were seeded per well in 24-well plates and incubated at 37 °C for 24 h. The CM was then removed and the PCM-1000 (hereafter PCM) was added in the absence or presence of the described concentrations of the CaSR agonist. The cultures were then incubated for 24 h, after which PrestoBlue™ end-point analyses were conducted as described above.

### 2.7. CaSR Antagonist, NPS2143 Hydrochloride

One millimolar stock solutions of NPS2143 hydrochloride (selective CaSR receptor antagonist, purity ≥99%, purchased from Tocris, Abingdon, UK) were prepared in dimethyl sulphoxide (DMSO). Further dilutions were prepared in culture medium (CM). The culture medium was removed from the MG63 cells and replaced with fresh CM containing NPS2143 hydrochloride or just CM for cultures not treated with the antagonist. The cells were then incubated for 2 h at 37 °C. After incubation, the culture medium was removed from all cells and replaced with CM, PCM, and NPS2143 hydrochloride, as appropriate. The cell cultures were then incubated for a further 22 h incubation at 37 °C, after which PrestoBlue™ end-point analyses were conducted as described above.

### 2.8. Measurement of Gene Expression

MG63 cells were seeded at a density 50,000 cells/well in 24-well plates and incubated for 24 h at 37 °C. The culture medium (CM) was then removed from the cells and either fresh control medium or PMC was added to the cells and the cultures were incubated for 1, 3, 5, and 7 days at 37 °C.

On days 3 and 5, the culture media were removed and replaced with fresh CM or PMC. After the required period of incubation, the media were removed and the cultures were washed with PBS, after which the cell monolayers were trypsinised and the cells were pelleted by centrifugation. RNA was extracted using ISOLATE II RNA Mini Kit (Bioline, Distributor: Scientific Laboratory Supplies, Nottingham, UK) and the RNA concentration was measured using a Nanodrop spectrophotometer (Thermo Fisher Scientific, Paisley, UK). Five hundred nanograms of total RNA for each condition was then reverse transcribed to cDNA using the High Capacity cDNA Reverse Transcription Kit (Applied Biosystems, Thermo Fisher Scientific, Paisley, UK) in a thermal cycler. Initial denaturation was carried out at 25 °C for 10 min followed by annealing at 37 °C for 120 min and a final elongation at 85 °C for 5 min, after which the temperature was reduced to 4 °C. The human gene sequences chosen for this study to quantify osteogenic differentiation were ALPL (alkaline phosphate), COL1A1 (collagen 1 alpha 1), BGLAP (osteocalcin), and RUNX-2. Real-time RT-PCR (qPCR) reactions were set up with 0.5 μL cDNA, TaqMan Master Mix (Applied Biosystems, Thermo Fisher Scientific, Paisley, UK). 3-Glyceraldehyde phosphate dehydrogenase (GAPDH, primer sequence 4326317E, Applied Biosystems, Thermo Fisher Scientific, Paisley, UK) was used as an endogenous control, and the corresponding commercial primer sequences [60,61], namely ALPL (Hs01029144_m1, Applied Biosystems, Thermo Fisher Scientific, Paisley, UK), COL1A1 (Hs00164004_m1,), BGLAP (Hs01587814_g1, Applied Biosystems, Thermo Fisher Scientific, Paisley, UK), and RUNX-2 (Hs00231692_m1,Applied Biosystems, Thermo Fisher Scientific, Paisley, UK) were added to a total reaction volume of 10 μL. The qPCR was carried out using the Applied Biosystems 7900HT Fast RT PCR system, (Thermo Fisher Scientific, Paisley, UK) and consisted of 40 cycles (95 °C for 15 s, 60 °C for 30 s, and 72 °C for 40 s) after an initial denaturation step (50 °C for 2 min). Initial analyses were carried out using the 7900HT Fast RT PCR system software, RQ Manager Software version 1.2. (Applied Biosystems, Thermo Fisher Scientific, Paisley, UK) to normalise and express the detection values of the target gene in relation to GAPDH values. The ΔΔCt method was used to calculate gene expression normalised to GAPDH values.

## 3. Results

### 3.1. Effect of nHA Paste-Conditioned Medium on Cell Metabolic Activity

**Figure 1:**
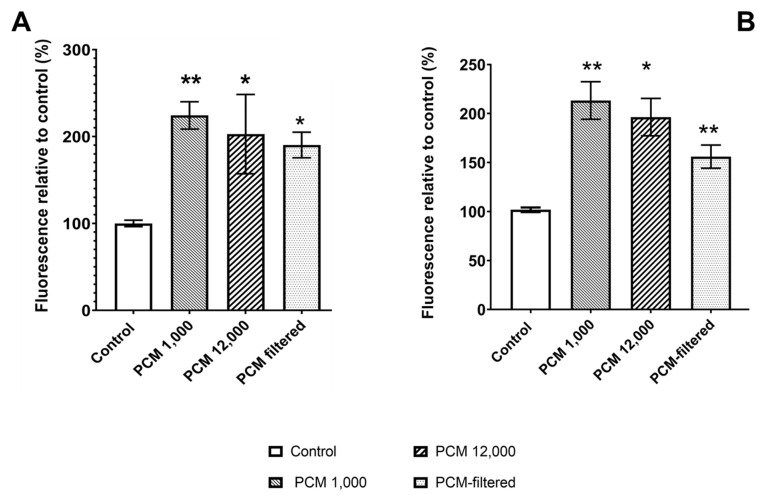
Effect of culturing (**A**) MG63 and (**B**) Saos-2 cells for 24 h in CM (culture medium), PCM-1000 (paste conditioned medium centrifuged at 1000 g), PCM-12000 (paste conditioned medium centrifuged at 12,000 g), or PCM-0.22 (paste-conditioned medium filtered through a 0.22 µm filter). Cell viability/activity was determined using PrestoBlue™. Bars indicate the mean percentage fluorescence values (±SD, n = 3) relative to the cells incubated in culture medium. * = *p* ≤ 0.05, ** = *p* ≤ 0.01.

The preconditioned culture medium was prepared in three ways: using either centrifugation at 1,000 g (PCM-1000) or 12,000 g (PCM-12000) or filtration through a 0.22 µm filter (PCM-0.22). The two cell lines studied (MG63 and Saos-2) showed a stimulated cell activity for all the PCMs, although the magnitude of the stimulation was different according to the method used to prepare the conditioned medium (Figure 1). For both cell lines, the highest metabolic activity was found for cells cultured in PCM-1000 and the lowest metabolic activity was found for cells cultured in PCM-0.22. PCM-1000 (PCM) was used in all subsequent experiments.

### 3.2. Cell Metabolic Response of Human Osteoblastic Cells, hBM-MSCs, and Murine Myoblastic Cells to nHA paste-Conditioned Medium (PCM)

**Figure 2:**
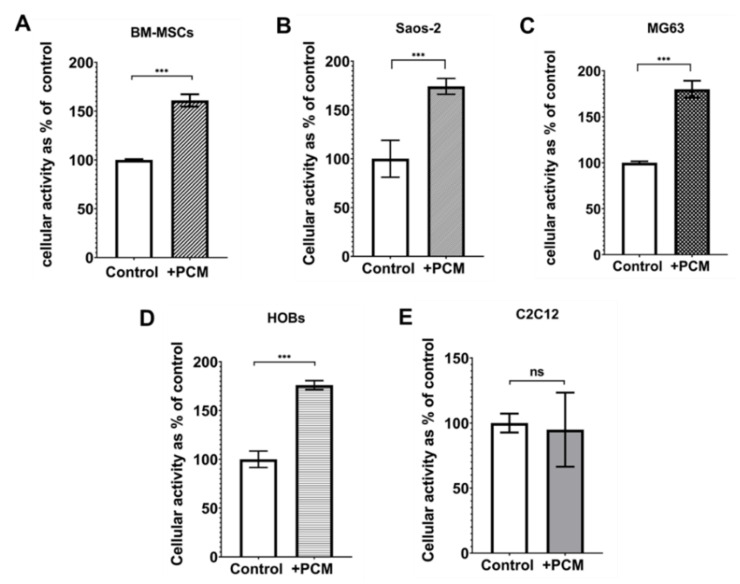
The levels of cell activity in human cells of osteoblastic lineage (MG63 and Saos-2), primary osteoblasts (HOBs), and BM-MSCs cultured with PCM-1000 or CM for 24 h. Cell activity was determined using PrestoBlue cell viability reagent. (**A**) hBM-MSCs, (**B**) Saos-2 cells, (**C**) MG63 cells, (**D**) HOBs, and (**E**) C2C12 cells. Bars indicate the mean level of cellular activity normalised to that of the control cell cultures (±SD, n = 3) incubated in CM (values are the means ± SD, n = 3, *** = *p* ≤ 0.0005, ns = no significant difference between samples).

The metabolic activity of HOBs, hBM-MSCs, MG-63, Saos-2, and C2C12 cells incubated for 24 h with nHA paste-conditioned medium (PCM) was compared with that of cells incubated in culture media (CM) alone (Figure 2). These results showed a significant difference in the metabolic activity between cells cultured in PCM and cells cultured in CM. The metabolic activity of all of the osteoblastic cells and the hBM-MSCs was stimulated by the nHA paste-conditioned media (PCM) compared to that of cells in culture medium only. PCM increased the metabolic activities of MG63 cells by 80% (Figure 2, image C), Saos-2 by 97% (Figure 2, image B), HOBs by 75% (Figure 2, image D), and hBM-MSC by 108% (Figure 2, image A) compared to the culture of the MG63 cells in CM alone. However, the PCM did not stimulate the cellular metabolic activity of the myoblastic C2C12 cell line (Figure 2, image E), where the metabolic activity of the PCM-incubated cells was 83% compared to that of the control C2C12 cells.

**Figure 3:**
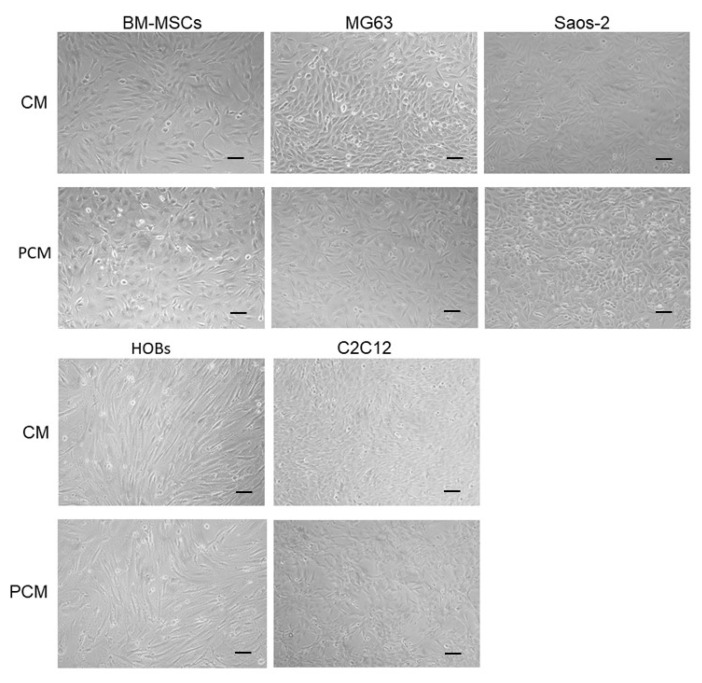
This figure shows phase-contrast images of BM-MSC, MG63, Saos-2, HOBs and C2C12 cell cultures after a24 h incubation with CM or PCM. Scale bars = 100 µm.

Figure 3 shows phase-contrast images of the osteoblastic cells, BM-MSCs, and C2C12 before and after a 24 h incubation in PCM or medium only. No disruption to the cell monolayers, cell lysis, or significant morphological changes were observed in any of the cell types after the incubation with PCM. 

### 3.3. Osteogenic Potential of the PCM

**Figure 4:**
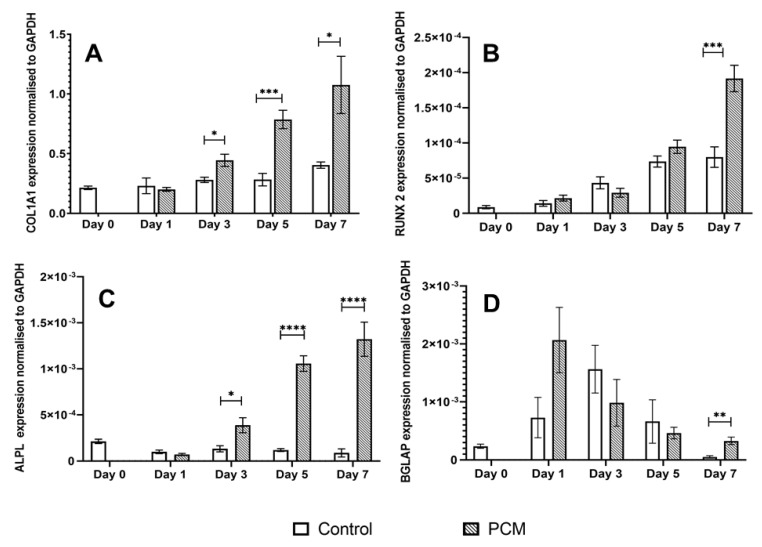
Gene expression of MG63 cells cultured in CM and PCM for (**A**) COL1A1, (**B**) RUNX-2, (**C**) ALPL, and (**D**) BGLAP genes. Results are shown normalised to GAPDH. * = *p* ≤ 0.05, ** = *p* ≤ 0.005, *** = *p* ≤ 0.0005, **** = *p* ≤ 0.00005.

RT-qPCR was used to quantify the levels of gene expression of alkaline phosphatase (ALPL), collagen 1 alpha 1 (COL1A1), osteocalcin (BGLAP), and RUNX-2 genes in MG63 cells in response to incubation of the cells with PCM. PCM-treated cells showed a marked increase in the gene expression of ALPL (Figure 4C) and COL1A1 (Figure 4A) from day 3 to day 7. In contrast, cultures incubated in CM did not show any marked increase in ALPL or COL1A1 expression over the 7-day incubation period. The expression level of RUNX2 (Figure 4B) in control cultures (cultured in CM) was observed to increase gradually from day 1 to day 7. PCM was only found to stimulate RUNX-2 gene expression above that in CM on day 7. In contrast, PCM stimulated BGLAP (osteocalcin) gene expression (Figure 4D) with a peak level of expression after 1 day of culture which declined from day 3 to day 7. The time course of PCM-stimulated gene expression was different to that of cells cultured in CM where the peak of BGLAP gene expression occurred on day 3 and then declined over days 5 and 7.

### 3.4. Determination of Ca^2+^ and Na^+^ Ion Concentrations in the PCM Samples

Analysis of the PCM and CM by ICP-OES showed that the Na^+^ ion concentrations in the PCM and CM were very similar (Table 1). ICP-OES could not accurately determine Ca^2+^ in the PCM as the Ca^2+^ concentration was below the 10 mg/L limit of detection of the analyser. Analysis of the PCM using the Ca^2+^ calorimetric assay showed the Ca^2+^ concentration to be significantly lower (*p* ≤ 0.0005) than that of the culture medium (Table 1).

### 3.5. Effect of Low Ion Concentrations on Cellular Metabolic Activity

**Figure 5:**
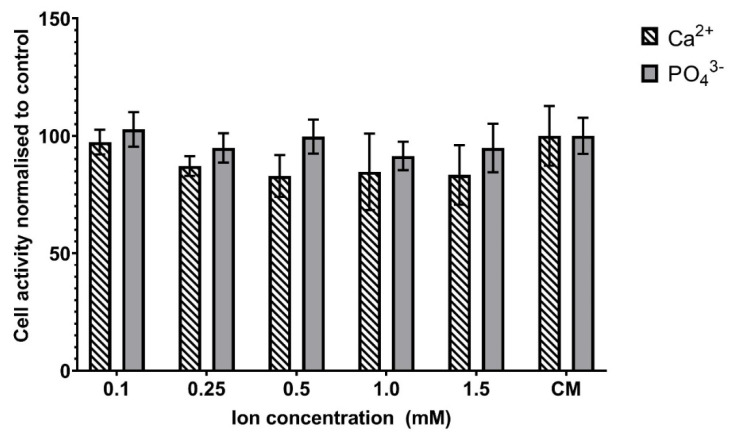
Effect of calcium and phosphate ions on the metabolic activity of MG63 osteoblastic cells. PrestoBlue analysis following the exposure of cells to varying concentrations of calcium and phosphate ions. Bars indicate the mean percentage fluorescence values (±SD, n = 3) relative to the fluorescence of cells incubated in normal culture medium (CM) only.

Figure 5 shows the effect of the addition of known, extracellular concentrations of Ca^2+^ and PO_4_^3−^ ions on the metabolic activity of MG63 cells. The calcium and phosphate ions were added in a range spanning the concentration above and below those measured in the PCM (Table 1). In contrast to the PCM, the addition of a range of Ca^2+^ or phosphate concentrations between 0.1 and 1.5 mM did not cause an increase in the metabolic activity of the MG63 cells (Figure 5). For both ions, there was no significant difference between the cell activity of the control and cells incubated with 0.1 to 1.5 mM calcium or phosphate ions. The addition of higher concentrations of calcium to the culture medium significantly stimulated the metabolic activity of MG63 cells with percentage increases in cell activity over that of control cells of 40.49 ± 17.5% (5 mM Ca^2+^, *p* ≤ 0.05), 49.2 ± 17.5% (10 mM Ca^2+^, *p* ≤ 0.05), and 102.0 ± 13.8% (20 mM Ca^2+^, *p* ≤ 0.001). The same concentration range of sodium ions caused no significant change in the cellular metabolic activity of the cells.

### 3.6. Effect of PCM on the Calcium-Sensing Receptor (CaSR) of Osteoblastic Cells

**Figure 6:**
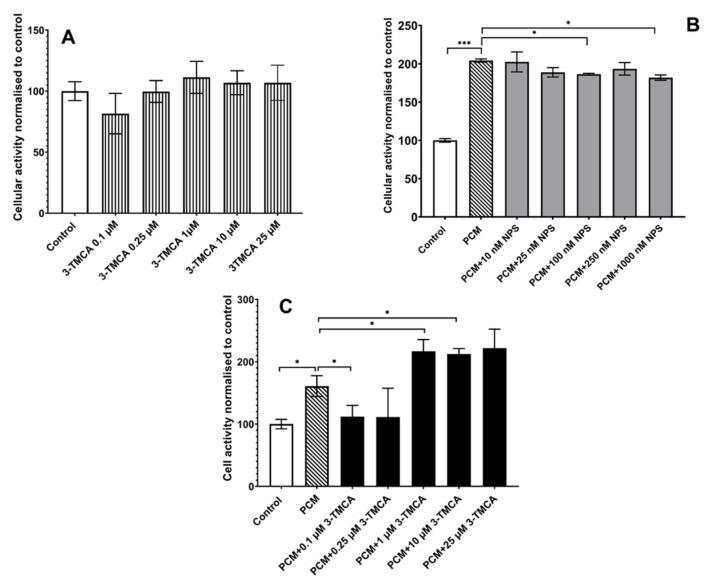
Effect of increasing concentrations of the calcium-sensing receptor agonist 3-TMCA and antagonist NPS 2143 on the cell activity of MG63 cells. Cell activity was assessed using PrestoBlue as described in Section 2. (**A**) Effect of 3-TMCA alone, (**B**) effect of increasing concentrations of NPS 2143 hydrochloride co-incubated with PCM, and (**C**) effect of PCM co-incubated with increasing concentrations of 3-TMCA on the metabolic activity of MG63 cells. Bars indicate the mean cell activity shown as the percentage fluorescence values (±SD, n = 3) relative to the control cells incubated in culture medium (CM) only. * = *p* ≤ 0.05, *** = *p* ≤ 0.0005.

3-(Trifluoromethyl)cinnamic acid (3-TMCA) is a member of a family of compounds known as calcimimetics. 3-TMCA is a CaSR agonist which binds to the receptor to increase the sensitivity of the CaSR to calcium. NPS2143 hydrochloride is a selective CaSR antagonist. Four different experimental conditions were studied: (i) cells incubated with CM, (ii) cells incubated with PCM, (iii) cells incubated with different concentrations of the CaSR agonist or antagonist, and (iv) cells cultured with PCM and different concentrations of the CaSR agonist or antagonist. In contrast to PCM, MG63 cells cultured in CM with 3-TMCA (Figure 6A) did not show an increase in cellular metabolic activity, whereas MG63 cells cultured in PCM did show increased cell activity. These results indicated that activation of the CaSR did not cause the increased cellular metabolic activity induced by the PCM. Further, the addition of the CaSR antagonist, NPS2143 hydrochloride, did not inhibit the PCM-induced stimulation of the cellular metabolic activity (Figure 6B). However, when cells were cocultured in PCM and concentrations of 3-TCMA (Figure 6C) of 1 µM and above, the cell metabolic activity increased significantly above that observed with PCM alone (*p* ≤ 0.05). These results suggested that stimulation of the CaSR enhanced the effects of the PCM on the metabolic activity of the MG63 cells.

**Figure 7:**
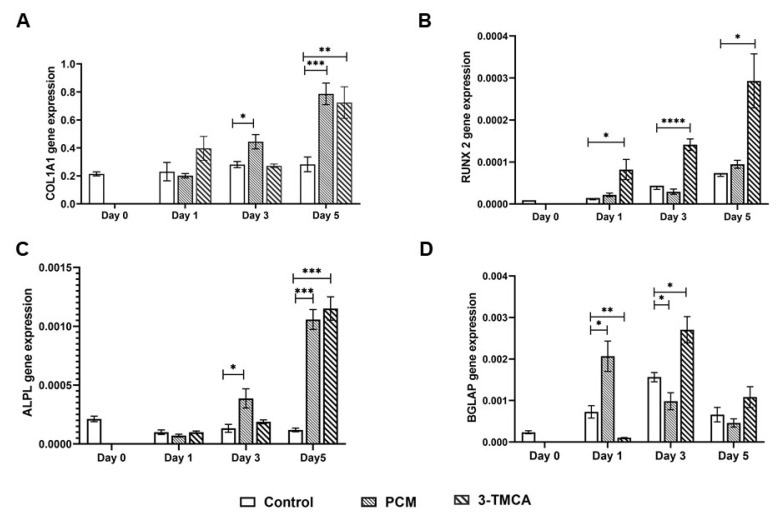
Comparison of the effects of PCM and the calcium-sensing receptor agonist 3-TMCA (1 µM) on expression of osteogenic genes in MG63 osteoblastic cell line. (**A**) COL1A1 (collagen I), (**B**) RUNX-2, (**C**) ALPL (alkaline phosphatase), (**D**) BGLAP (osteocalcin). Results are shown normalised to GAPDH and the day 0 cell controls. * = *p* ≤ 0.05, ** = *p* ≤ 0.005, *** = *p* ≤ 0.0005, **** = *p* ≤ 0.00005.

Both PCM and the CaSR agonist 3-TMCA significantly stimulated osteoblastic gene expression in MG63 cells (Figure 7). Both agents significantly stimulated ALPL (alkaline phosphatase), COL1A1 (collagen 1 gene,), BGLAP (osteocalcin gene), and RUNX-2 gene expression. However, the magnitude of response and time course of stimulation differed between the PCM and 3-TMCA. The CaSR agonist gave much higher stimulation of RUNX-2 gene expression than the PCM at days 1, 3, and 5 (Figure 7B). Additionally, the time course of the CaSR agonist-stimulated BGLAP gene expression was different to that of the PCM (Figure 7D). 3-TMCA significantly stimulated BGLAP-gene expression on days 3 and 7 only with a peak BGLAP expression at day 3. In contrast, the PCM showed a peak of BGLAP gene expression on day 1 which declined in magnitude on days 3 and 7. The time course of COL1A1 and ALPL gene expression was similar for both the PCM and 3-TMCA (Figure 7A,C). Both agents showed a significant increase in gene transcription on day 3 with a further increased expression on day 5.

## 4. Discussion

Previous studies on the biocompatibility of nHAs have demonstrated the important roles played by particle size [25] and shape [24] in terms of potential cell toxicity caused by cellular uptake of nanoparticles. Studies with hepatocytes [29] showed that nHA uptake by cells induced cell death through damage sustained by the cells through (i) accumulation of nHA-filled endosomes or (ii) degraded endolysosomes/lysosomes in the cytoplasm. Therefore, an indirect contact toxicity assessment method based on the ISO 10993-5:2009 standard guidelines was used in this study to investigate nHA-PCMs prepared by centrifugation (1,000g or 12,000g) or filtration through a 0.22 µm filter of the medium collected from incubation of the nanopaste with culture medium (CM). The centrifugation and filtration were used to remove large aggregates of nanoparticles.

The viability data showed that cellular metabolic activity was increased around 2-fold with the PCMs in all the osteoblastic cells tested. The PCM also enhanced the metabolic activity of hBM-MSCs, which are the stem cell phenotype from which the osteogenic precursors usually develop in vivo. However, in this study, the PCM-induced increase in cell metabolic activity was restricted to bone-derived cells as it was not seen in C2C12 cells, which are a myoblast cell line and not an osteoblastic lineage. The PCM did not induce any disruption to the cell monolayers, cell lysis, or significant morphological changes in any of the cell types tested. Together, the cell viability data and the microscopic images show that the nHA PCM was biocompatible with all the cell types tested.

It is well known that calcium ions have an important role in many different cellular functions such as secretion, apoptosis, and the regulation of proliferation [45]. The normal calcium concentration in the plasma is in a narrow range of 2.1 to 2.6 mM with an ionised calcium concentration of 1.1 to 1.4 mM [62]. The Ca^2+^ ion concentration of the PCM was determined to be 0.45 ± 0.04 mM compared to the 1.8 mM Ca^2+^ of the culture media. The reduction in the free Ca^2+^ concentration in PCM is likely to be attributable to solution-mediated surface reactions between the nHA and Ca^2+^ ions in the culture medium, indicating the nHA paste was a calcium-deficient hydroxyapatite [61,63]. In addition, instability of nHA in the culture medium can induce the formation of nHA agglomerates [27], which could be pelleted during centrifugation of the PCM. However, further studies are needed to confirm this point.

The PCM significantly stimulated metabolic activity in the osteoblast-lineage cells and BM-MSCs. This effect was not mimicked by CM containing 0.1 to 1.5 mM Ca^2+^, i.e., a range of Ca^2+^ concentrations similar to the 0.45 mM Ca^2+^ in the PCM. This result indicated that the Ca^2+^ ion concentration of the PCM was too low to directly stimulate the metabolic activity of the cells. However, the cell activity of MG63 cells was significantly stimulated by CM containing high (5 to 20 mM) levels of calcium. These extracellular calcium concentrations are similar to those reported to stimulate osteoblast proliferation (2–6 mM) and optimal cell differentiation (6–10 mM) [61,64,65,66]. Additionally, 5 mM Ca^2+^ and above could activate the CaSR on the cells [40,67]. In summary, the results strongly indicated that calcium ions and plausibly nanoparticles of nHA released from the paste positively influenced cell metabolism and that this effect was specific to bone-derived cells, i.e., cells of the osteoblastic lineage and BM-MSCs.

Real-time RT-PCR showed that the PCM significantly stimulated the expression of osteogenic genes ALPL, COL1A1, osteocalcin, and RUNX-2 in the MG63 osteoblastic cells. The gene transcription of ALPL, COL1A1, and RUNX-2 increased from day 3 to day 7 of the incubation period. In contrast, BGLAP expression (osteocalcin gene) peaked at day 3 then decreased during the remaining incubation time. This PCM-stimulated osteogenic gene expression occurred although the PCM had a low extracellular Ca^2+^ ion concentration of 0.45 ± 0.049 mM. Duvorak et al. [68] have reported stimulation of the osteocalcin gene by 0.5 mM extracellular Ca^2+^ ions, which is close to the Ca^2+^ of the PCM. These findings strongly supported an osteogenic effect of the nHA paste, increasing confidence in a previous report [17] that the commercial nHA paste Ostim stimulated BMP and VEGF-mediated signal pathways in human subjects. Pilloni [69] has also reported increased gene expression of BMP 5, BMP 7 and COL1A2 in human primary alveolar osteoblasts at the end of a 14-day culture period on poly-L-lysine-nHA substrate. In contrast are several reports which show that incubation of BM-MSCs with nHAs causes a rapid reduction in ALPL and OPN (osteopontin) gene expression [70,71]. In these studies, the BM-MSCs are stimulated to directly differentiate in osteoblasts (intramembranous ossification route) while inhibiting differentiation into chondrocytes and endochondral ossification [72].

The CaSR has a recognised important role in bone development, mineralization, and bone turnover [40]. Stimulation of osteogenic responses by calcium ions has been correlated with activation of the CaSR [73]. Sarem et al. have reported a hyperstimulation of the CaSR in human MSCs by a biomimetic apatite [72] to influence MSC differentiation fate down an osteoblastic rather than chondrocytic pathway. The data presented here show that the stimulation of cell activity by the PCM in osteoblastic cells (MG63 cells) was not inhibited by the addition of a known CaSR antagonist, NPS2143 hydrochloride [73]. However, it is acknowledged that effective suppression of CaSR activity can be difficult [53]. The stimulation of cell activity of MG63 cells was not replicated by incubation with the allosteric CaSR agonist 3-TMCA. This lack of effect by the CaSR agonist and the inability of the CaSR antagonist to inhibit PCM-stimulated cell activity strongly indicated that the PCM enhanced the metabolic activity of osteoblastic cells by a CaSR-independent mechanism.

Addition of low concentrations of 3-TMCA to PMC-stimulated cells inhibited the stimulation of cell activity in the MG63 cells. This apparent aberrant effect could be explained if there was competition of the intracellular pathways activated by the 3-TMCA-activated CaSR and PCM for Ca^2+^ ions. Additionally, higher concentrations of 3-TMCA enhanced PCM-stimulated cell activity in the MG63 cells. These data could suggest that greater activation of the CaSR by 3-TMCA increased the intracellular Ca^2+^ ions sufficiently [74] so that further activation of the PCM intracellular pathway occurred. Further experiments are required to support these explanations for the effects of 3-TMCA in PCM. Suto et al. [75] have reported that HA nanoparticles may not release Ca^2+^ or PO_4_^3−^ ions extracellularly but act via cellular internalisation of nanoparticles. This mechanism could also explain the results obtained with the CaSR agonist and antagonist presented in this paper. The addition of the CaSR agonist 3-TMCA also significantly stimulated osteoblastic gene expression (COL 1A1, ALPL, RUNX-2, and BGLAP). However, the time course of peak 3-TMCA stimulation of BGLAP occurred on day 3 compared to day 2 for the PCM. The time course of stimulation of RUNX-2 also was different to that of PCM. Overall, these data suggested that the stimulation of osteogenic gene transcription by the CaSR may be occurring by a different intracellular mechanism to the PCM-stimulated cell activity in the MG65 cells.

3-TMCA had no significant effect on the metabolic activity of MG63 cells in control culture medium in the absence of PCM. These results can be explained if the calcium ion concentration of the nHA PCM was too low to stimulate the CaSR directly and the PCM enhanced the cellular metabolism of the MG63 cells by a CaSR-independent mechanism. On addition of 3-TMCA to the MG63 cells in PCM, the allosteric activation of the CaSR receptor by 3-TMCA enabled it to respond to extracellular Ca^2+^ions in the order of 0.5 mM [49] as found in the PCM. Therefore, 3-TMCA produced a further increase in metabolic activity through a CaSR-dependent mechanism in addition to the CaSR-independent PCM mechanism. Although the intracellular mechanism of action of nHAs remains to be fully evaluated, the data presented in this study strongly support a mechanism that does not require stimulation of the CaSR. The data reported in this paper are not in opposition to those of Sarem et al. [72] discussed above. This paper reports the effects of nHA on differentiated osteoblastic cells, whereas Sarem et al. investigated the effect of a biomimetic nHA on the differentiation of MSCs into osteoblasts.

To summarise, the nHA investigated here was found to be not cytotoxic, but instead was biocompatible in that it stimulated biological processes that were related to natural bone healing. This counters a number of previous studies, where either the cells were not of osteoblastic lineage or there were limitations associated with the methodological approach. Very little is known about the role of the CaSR in the intracellular mechanism of nHAs. This study has demonstrated that the CaSR was not directly involved in the action of the nHA paste on osteoblastic cells, which is contrary to the effect of nHA reported on MSC differentiation [72]. The study reported here has important implications for the translation of nanotechnology from the laboratory to clinical adoption, and it may be of additional value to regulators seeking to understand the data presented in support of market approval for these types of medical devices in the future.

## 5. Conclusions

Previous clinical and animal studies have shown that nHA promotes bone-tissue regeneration in vivo, but little was previously understood regarding the underlying mechanisms. Moreover, there were generally held concerns regarding the biocompatibility of nanoparticles. In this study, an indirect method for assessing toxicity was developed to investigate the effect of bioinspired nHA particles on cell metabolic activity. This investigation showed that nHA-conditioned medium (PCM) increased metabolic activity in primary osteoblasts, MSCs, and osteoblastic cell lines. In osteoblastic cells, the PCM also stimulated osteogenic gene transcription. It was concluded that the nHA paste investigated here has osteogenic properties, explaining previous reports of tissue regeneration in a critical-size surgical defect ovine model [12]. Moreover, the data presented suggest that cell responses to nHA vary not only according to the morphology of the nanoparticle, but also the type of cell used in the study. Bone cells responded positively to nHA, and this research has added considerably to the understanding of the mechanisms responsible for excellent biocompatibility and stimulation of bone-tissue healing.

## Figures and Tables

**Table 1:** Concentrations of sodium and calcium ions in culture medium (CM) and medium conditioned with the nHA paste (PCM). Values are the means ±SDs of triplicate samples (*** = *p* ≤ 0.0005). The theoretical concentrations of the two ions in the CM (according to the medium formulation) are also shown in italic font.

Sample	Na^+^ (mM)	Ca^2+^ (mM)
*CM (theoretical values)*	*143.5*	*1.80*
CM	154.30	1.85 ± 0.04
PCM	133.40	0.45 ± 0.04 ***

## Data Availability

Not applicable.
